# Supplementary material for: Repurposing Niclosamide as a Novel Anti-SARS-CoV-2 Drug by Restricting Entry Protein CD147
Source: Biomedicines. 2023 Jul 18;11(7):2019. doi: 10.3390/biomedicines11072019 (PMC10377517; doi:10.3390/biomedicines11072019)
Supplement: Supplementary file 1 [file biomedicines-11-02019-s001.zip › biomedicines-2473171-supplementary.pdf]

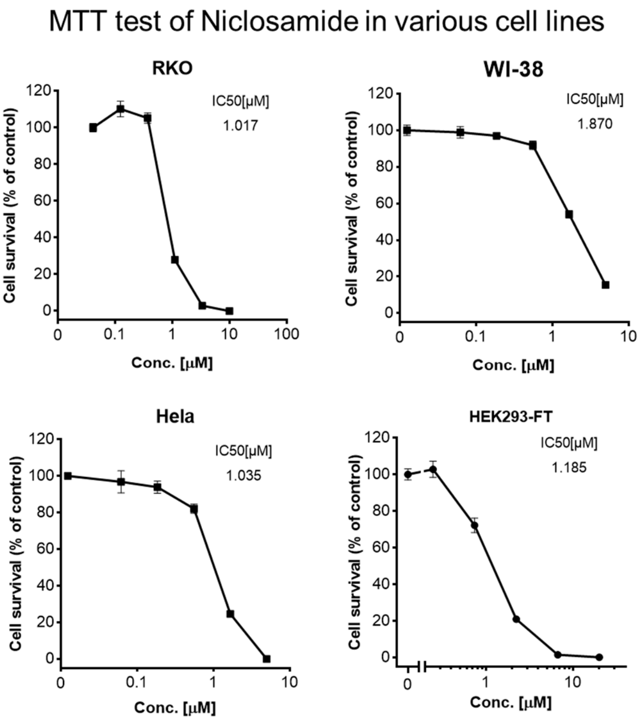

Figure S1: MTT test of niclosamide in various cell lines.
